# Prevalence and Predictive Factors of Compassion Fatigue among Healthcare Workers in Saudi Arabia: Implications for Well-Being and Support

**DOI:** 10.3390/healthcare11152136

**Published:** 2023-07-26

**Authors:** Ahmad H. Almadani, Shuliweeh Alenezi, Maha S. Algazlan, Ebraheem S. Alrabiah, Reem A. Alharbi, AlRabab S. Alkhamis, Mohamad-Hani Temsah

**Affiliations:** 1 Department of Psychiatry, College of Medicine, King Saud University, Riyadh 11362, Saudi Arabia; 2 Department of Psychiatry, King Saud University Medical City, King Saud University, Riyadh 11362, Saudi Arabia; 3 Eradah Complex and Mental Health, Buraydah 52366, Saudi Arabia; 4 Department of Psychiatry, College of Medicine, Imam Abdulrahman Bin Faisal University, Dammam 31441, Saudi Arabia; 5 Pediatric Department, College of Medicine, King Saud University, Riyadh 11362, Saudi Arabia; 6 Prince Abdullah bin Khaled Coeliac Disease Research Chair, King Saud University, Riyadh 11362, Saudi Arabia

**Keywords:** prevalence, predictive factors, compassion fatigue, healthcare workers, Saudi Arabia, well-being, support, burnout, secondary traumatic stress, professional quality of life

## Abstract

Compassion fatigue (CF) poses significant challenges to healthcare workers’ (HCWs) well-being. This study aimed to estimate the prevalence of CF and identify its predictive factors among HCWs in all regions of Saudi Arabia (SA). As such, all HCWs from different disciplines in different centers were allowed to participate, resulting in 678 participants. The study tool, distributed between October 2022 and January 2023, consisted of a questionnaire created by the authors based on the Professional Quality of Life Scale (ProQOL). The ProQOL measures the positive (compassion satisfaction [CS]) and negative (CF) effects of helping those who have suffered, noting that burnout (BO) and secondary traumatic stress (STS) are the two subscales that constitute CF. Our findings revealed that 63.9% of HCWs experienced average STS, while 57.2% reported average BO levels. HCWs in the southern and northern regions exhibited higher STS (*p*-value = 0.003 and 0.010, respectively). Physicians displayed higher BO levels (*p*-value = 0.024). Higher levels of CS were found among older HCWs (*p*-value = 0.001) and lower levels among those with more years of experience (*p*-value = 0.004). Support at work and job, life, and financial income satisfaction were significantly and positively correlated with CS and negatively correlated with BO and STS. These findings highlight the need for tailored awareness campaigns targeting HCWs, particularly physicians, to promote well-being, enhance coping skills, and foster problem-solving techniques. Keywords: burnout; compassion fatigue; compassion satisfaction; healthcare workers; professional quality of life; Saudi Arabia; secondary traumatic stress; medical trainees’ well-being.

## 1. Introduction

Healthcare workers (HCWs) inherently display compassion while caring for patients, which can lead to satisfaction [1]. Helping patients who are suffering (or the desire to help) might come at a high cost, however, and have significant negative consequences, including compassion fatigue (CF) [2]. CF is a work-related psychosocial consequence that may result from exposure to a cumulative level of trauma and dealing with those who have been traumatized [3]. It is associated with severe emotional distress, desensitization to patients’ suffering, lack of passion for patient care, and adverse clinical outcomes [4,5,6].

Another challenge HCWs face is burnout (BO), which is the weariness and discontent resulting from the demanding and stressful work environment [7]. A term related to CF and BO is secondary traumatic stress (STS). STS is an extreme response to traumatic stressful events or patients who are traumatized. Extreme responses of STS include intrusive images, avoidance of reminders of traumatic experiences, fear, and sleep disturbances [8]. CF, BO, and STS are closely related concepts and are sometimes used interchangeably, but they are distinct [9]. CF, BO, and STS could lead HCWs to consider leaving their profession [10].

Compassion satisfaction (CS) is a favorable outcome, unlike CF, BO, and STS. It is often described as the sense of pleasure and satisfaction that HCWs feel from caring for sick patients [4]. Several elements play a role in CS, one of which is the level of support from coworkers [11]. CF, BO, and CS are three variables that might affect the professional quality of life of health professionals, which refers to the positive and negative aspects of a job that involves helping others who experience trauma and suffering [12]. CF positively correlates with BO but negatively with CS [4,13].

Multiple studies have examined CF, BO, and CS in various settings and demographics. Among physicians, most emergency medicine consultants reported average CS, and those who reported low scores were more likely to intend to retire early [14]. In pediatric palliative care professionals in the US, CF was found to affect personal well-being and professional effectiveness [15]. The influence of other factors has also been studied. In maternal-fetal medicine, physicians, female gender, self-report of significant emotional deficit, and use of mental health services were significant predictors of higher CF [16]. In the nursing field, nurses with more years of experience, 16 to 20 years, had less STS than other nurses who had less experience [17].

During the COVID-19 pandemic, the quality of life of health professionals was significantly affected. For instance, a systematic review addressed the fact that BO and CF levels increased during the pandemic. Anxiety, depression, insomnia, and sociodemographic variables such as female gender, the nursing profession, and working directly with COVID-19 patients were considered risk factors for developing BO. Similarly, being female and working with COVID-19 patients were associated with higher scores of CF. Resilience and social support were found to be protective against BO [18]. Another study evaluated BO among HCWs in two phases one year apart. In the study’s first phase, higher BO levels were observed in females, those under 30, those who did not have children, and those who were postgraduates. In the second phase, the psychological suffering was no longer markedly concentrated in specific bands of HCWs, which suggests more widespread distress during the pandemic [19].

Only a few studies have been conducted in Saudi Arabia (SA). A cross-sectional study in four Saudi public hospitals showed that working hours, educational levels, and nationality were primary contributors to work-related stress and CF among Saudi Arabian nurses during the COVID-19 pandemic [20]. Another cross-sectional study was conducted in emergency departments of public hospitals in Dammam City. Most emergency physicians and nurses reported high levels of CF and low personal achievements. The leading causes of high BO were being male, being a married woman, having poor relationships with colleagues, working more hours, having fewer rest days, and suffering from psychiatric disorders [21]. A recent study was conducted to evaluate CF among psychiatrists and psychiatry trainees in SA using the Professional Quality of Life Scale. The findings indicated that 65% of respondents reported an average level of BO, 43% reported an average level of CF, and only 39% reported a high level of CS. Female gender was related to higher BO. Having a personal trauma history was associated with high CF, and divorce or separation was linked with high CS [22].

To date, CF has been studied in healthcare providers in a limited range of settings in Middle Eastern countries and more so in SA. Moreover, to our knowledge, research in SA has yet to be undertaken to include all HCWs from different disciplines. This gap in the literature limits the ability to identify and implement interventions to support HCWs and promote their well-being, noting that the impaired well-being of physicians affects the quality of care and impacts the healthcare systems negatively [23,24,25]. Given the topic’s significance and the limited research in SA, this study aims to determine the prevalence of CF among Saudi Arabian HCWs and explore contributing sociodemographic and work-related factors. This research could enrich the literature and guide future interventions. We hypothesize that CF is highly prevalent among HCWs in SA and that correlations between CF and demographic and practice characteristics are likely to be found, as previously suggested in various studies in the literature.

## 2. Materials and Methods

### 2.1. Study Design, Setting, and Participants

A quantitative cross-sectional study was conducted among HCWs in SA. The study tool was distributed electronically among the participants between October 2022 and January 2023 via the researchers’ professional networks and their social media channels, such as WhatsApp groups. The survey link was distributed online to reduce costs, to save time, and for easier geographical reach [26]. The targeted population of the study included all HCWs from all disciplines in different healthcare settings all over SA, including staff and trainees. According to the Ministry of Health, HCWs in SA are estimated to number around 485,688 [27]. Using the Raosoft sample size calculator, the required sample size was 384, with a 95% confidence interval and a margin of error of 5% [28].

This study used a convenience sampling approach. The participants in this study were grouped into (1) doctors, (2) nurses, and (3) allied HCWs. The inclusion criteria consisted of all healthcare workers (HCWs) working in different regions of Saudi Arabia (SA), including the central, eastern, western, northern, and southern areas at the time of data collection. The exclusion criteria were non-HCWs or those not working in SA at the time of data collection. The unit of analysis was the HCW.

### 2.2. Study Instrument

The study’s instrument consisted of two parts: (1) a questionnaire developed by the research team to gather sociodemographic, occupational, and personal clinical history information and (2) the Professional Quality of Life Scale version 5 (ProQOL 5) to measure CS and CF. The following data were gathered using the questionnaire: (1) demographic data about age, gender, nationality, marital status, having children or not, and region of employment in SA; (2) personal clinical history, including having a chronic medical or mental illness; and (3) practice information, such as disciplines, departments, years of experience, hospital levels (primary, secondary, or tertiary), service type (public or private), working hours per week, the approximate number of patients treated per week, and feeling supported or not. CS and CF were measured using ProQOL 5, one of the most-used tools in studying CF [8,29]. It is a 30-item self-report scale that evaluates both positive and negative aspects of caregiving. It consists of three subscales: CS, BO, and STS, noting that BO and STS subscales constitute CF. Each of the three subscales consists of 10 items that assess how frequently in the last 30 days a respondent has experienced symptoms. Respondents rate each item on a 5-point Likert scale (1 = never to 5 = very often). Each subscale produces a score that can be classified as low, moderate, or high, with scores outside the individual range suggesting a potential risk [8]. Thorough testing has been carried out on ProQOL 5, with results indicating high levels of reliability alphas for each phenomenon; CS demonstrated a reliability alpha of 0.88, while BO and STS showed alphas of 0.75 and 0.81, respectively, and it is a valid measure of each individual phenomenon [8].

### 2.3. Data Analysis

Continuous variables were described using mean and standard deviation statistics, while categorical variables were described using frequencies and percentages. The normality of metric variables was assessed using the Kolmogorov–Smirnov test and histograms. The internal consistency of the questionnaire was assessed using Cronbach’s alpha test. Correlations between metric variables were assessed using bivariate Pearson’s correlations. Multivariable linear regression analysis was performed to assess the association between predictor variables and the HCWs’ perceptions of the CF subscale scores, expressed as beta coefficients with associated 95% confidence intervals. Statistical analysis was performed using SPSS IBM version 21 (IBM Corp., Armonk, NY, USA) with a significance level of 0.050 [30].

## 3. Results

A total of 1085 HCWs gave their consent to participate in the study. Of those, 678 completed the survey, resulting in a completion rate of 62.5%. The remaining 407 responses were deemed invalid and eliminated by the research team due to incomplete data. As the exact number of individuals who received the survey link is unknown, it is impossible to determine the response rate.

**Figure 1:**
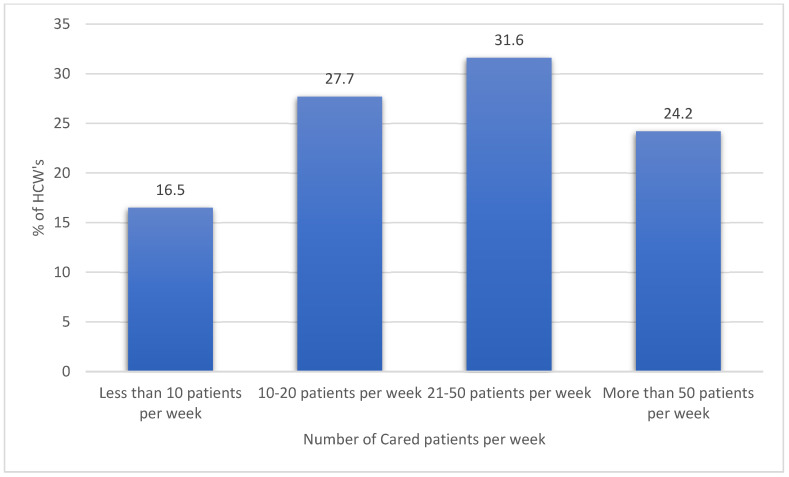
Healthcare workers’ number of cared patients per week.

Table 1 displays the resulting descriptive analysis of the HCWs’ sociodemographic characteristics, professional discipline, work setting, clinical experience, and workload. Most of the sample (63.3%) were female, and 36.7% were male. Moreover, the analysis findings showed that 51% of respondents were married, 40.4% had children, and most (74.5%) were Saudi citizens. The findings also showed that 62.1% of the participants resided in the central region of Saudi Arabia. Regarding the HCWs’ disciplines, 65% of them were physicians, 22.3% were nurses, and 12.7% were allied HCWs. Only a few (11.8%) had a history of psychiatric illness, and 20.2% had another chronic medical condition. The numbers of cared patients per week are shown in Figure 1. Also, the descriptive analysis for the HCWs’ perceived general life and work satisfaction and perceived CF indicators are shown in Table 2.

HCWs’ Satisfaction with Work and Life: The study found that the HCWs’ perceived satisfaction with support at their workplace and their job satisfaction were 2.95/5 and 3.16/5, respectively. The collective mean perceived satisfaction with their personal lives was 3.18/5, while their financial income satisfaction was 3/5 on average.

Perceived Burnout Indicators among HCWs: The top perceived indicators of BO among HCWs were their feeling of being very caring people (mean = 4.02/5), their sense of happiness (mean = 3.54/5), and their feelings of connectedness to others (mean = 3.48/5). However, the lowest perceived indicators of BO were loss of productivity due to sleep deprivation as a result of traumatic stress experiences associated with people they had helped (mean = 2.11/5), their feelings of being trapped by their jobs as helpers (mean = 2.55/5), and feeling worn out because of their jobs as helpers (mean = 3.01/5). The remainder of the BO indicators were rated midway between the top and lowest perceptions of BO.

Perceived Compassion Satisfaction Indicators among HCWs: The top perceived indicators of CS among HCWs were satisfaction from helping others (mean = 4.14/5), being proud of their ability to help (mean = 4.00/5), and liking their work as helpers (mean = 3.78/5). The lowest perceived indicators of CS were feeling invigorated after working with patients (mean = 3.30/5), feeling satisfied with their work (mean = 3.47/5), and feeling pleased with keeping up with helping techniques and protocols (mean = 3.51/5).

Perceived Secondary Traumatic Stress Indicators among HCWs: HCWs’ most-perceived STS indicators were having multiple patients to help (mean = 3.34/5), difficulty separating personal life from work as helpers (mean = 2.82/5), and being startled by unexpected sounds (mean = 2.66/5). The HCWs’ least perceived indicators of STS were having intrusive and frightening thoughts (mean = 2.28/5), feeling as if they were experiencing others’ trauma (mean = 2.32/5), avoiding situations that reminded them of trauma (mean = 2.32/5), and thinking that they might be affected by others’ traumatic stress (mean = 2.34/5).

Table 3 shows the descriptive analysis of HCWs’ perceived CF concepts and their perceived CF indicator levels. The mean score for perceived CS was 36.86/50, indicating a high CS overall: 2.8% of HCWs had low CS, 70.4% had average CS, and 26.8% had high CS. Perceived BO had a mean score of 23.40/50, with 42.5% of participants having low BO and 57.2% average BO. Perceived STS had a mean score of 25.57/50, with 33.9% having low STS, 63.9% average STS, and 2.2% high STS.

Table 4 displays the resulting bivariate correlations between the HCWs’ measured perceptions of work-related CF and other factors. The findings showed that the HCWs’ mean perceived BO correlated significantly, but negatively, with their mean perceived CS, r = −0.665, *p*-value < 0.01 (as the HCWs’ mean perceived BO tended to rise, their mean perceived CS tended to decline significantly on average). Additionally, the HCWs’ mean perceived STS score and their CS correlated negatively, but very weakly, r = −0.098, *p*-value < 0.05 (as the HCWs’ mean perceived STS tended to rise, their mean perceived CS score tended to decline incrementally on average). Moreover, the HCWs’ perceived support at work correlated positively and significantly with their mean perceived CS, r = 0.356, *p*-value < 0.010. Furthermore, their perceived job satisfaction correlated positively with their perceived CS score, r = 0.466, *p*-value < 0.01. In addition, their mean perceived life satisfaction and their mean perceived CS score converged significantly and positively, r = 0.403, *p*-value < 0.01. Nonetheless, satisfaction with financial income correlated significantly and positively with the HCWs’ perceived CS score, r = 0.268, *p*-value < 0.01. On the other hand, the HCWs’ mean perceived STS had a positive correlation with their mean perceived BO score, r = 0.445, *p*-value < 0.01. However, the HCWs’ mean perceived support at work correlated negatively with their mean perceived BO score, r = −0.418, *p*-value < 0.01, and their mean job satisfaction score correlated negatively with their mean perceived BO score, r = −0.445, *p*-value< 0.01. The HCWs’ mean perceived life satisfaction and their mean financial satisfaction scores both correlated negatively with their mean perceived BO score, *p* < 0.01 each.

The HCWs’ mean perceived received support at work score had a negative correlation with their mean STS score, r = −0.153, *p*-value < 0.01. Moreover, their mean perceived job satisfaction and general life satisfaction scores converged negatively on their mean perceived STS score, *p*-value < 0.01. In addition, the HCWs’ financial satisfaction mean score had a negative correlation with their mean perceived STS score, r = −1.02, *p*-value < 0.01. On the other hand, the HCWs’ mean perceived support at work and their mean perceived job, general life, and financial satisfaction scores all correlated significantly and positively with each other, with *p*-values of 0.01 each.

Multivariable linear regression analysis was applied to the HCWs’ mean perceived CS score to better understand why the HCWs perceived more or less satisfaction with compassion. Table 5 presents the results. These show that HCWs aged 41 or older had a significantly higher mean perceived CS score than those aged 40 or younger. The HCWs’ sex and marital status were not significantly correlated with their mean perceived CS score. However, HCWs with more than 5 years of experience had a significantly lower mean perceived CS score than those with less than 5 years of experience.

The study also found that the HCWs’ mean perceived job satisfaction score had a significant positive correlation with their mean perceived CS score, while their mean perceived BO score had a significant negative correlation. Moreover, the HCWs’ STS score was found to have a significant positive correlation with their mean perceived CS score after accounting for other predictor variables. The results suggest that improving job satisfaction and reducing BO and STS among HCWs could enhance their perceived CS and, subsequently, improve the quality of healthcare services.

Table 6 presents the results of a multivariable linear regression analysis examining the factors associated with HCWs’ perceived work-related BO. The analysis found that HCWs with children had significantly lower mean perceived work-related BO scores than those without children, while physicians had significantly higher mean perceived work-related BO scores than other HCWs. The HCWs’ region of residence did not significantly correlate with their perceived work-related BO score. The HCWs who worked more than 60 h per week had significantly higher mean perceived work-related BO scores than those who worked 60 h or less. In addition, the HCWs’ mean perceived STS at work had correlated positively and significantly with their mean perceived work-related BO scores. In contrast, the perceived level of support at work, perceived client satisfaction at work, and general life satisfaction were all significantly negatively correlated with work-related BO scores. Finally, HCWs with a history of mental illness had significantly higher perceived work-related BO scores than those without such a history.

Table 7 presents the results of the multivariable linear regression analysis for the mean perceived STS score among HCWs in SA. The analysis revealed that HCWs’ sex, age, having children, and marital status did not significantly correlate with their mean perceived STS score (*p*-value > 0.050). However, HCWs residing in the southern and northern regions of SA had significantly higher mean perceived STS scores than those residing in other regions (*p*-value = 0.003 and *p*-value = 0.010, respectively). 

In addition, HCWs’ mean perceived STS score had a positive correlation with their mean perceived CS score (beta coefficient = 0.368, *p*-value < 0.001), indicating that as their CS score increased, their predicted mean perceived STS score also tended to increase. Furthermore, the HCWs’ mean perceived work-related BO score had a positive correlation with their mean perceived STS score (beta coefficient = 1.063, *p*-value < 0.001), suggesting that higher BO perception among HCWs predicted significantly higher perceived STS on average.

## 4. Discussion

This is the first study to estimate the prevalence of CF and its associated factors that includes HCWs of all disciplines in SA. Our results indicated that more than half of the HCWs had average levels of STS, with only 2.2% of the participants having high STS. These results are aligned with previous studies [22,31] which reported average STS levels. Moreover, in our results, HCWs in both southern and northern SA perceived considerably greater STS. The reason for this finding might be that the southern and northern provinces of the country are proximate to war zones, which could increase the risk of exposure to more patient-related traumatic experiences.

Surprisingly, our results showed a positive correlation between CS and STS, which is inconsistent with a study on critical care nurses in Iran that found a negative association between CS and STS [17]. A possible explanation of our finding is that the satisfaction gained from helping others reflects high interest and care, resulting in more stress. Moreover, the results from our research demonstrated that greater STS correlates with increased levels of BO, which is consistent with a systematic review conducted to evaluate the impact of the COVID-19 pandemic on CF [18]. 

As identified in a study conducted on therapists in the UK (*n* = 253, who worked in trauma services, secondary-care services, or other services), our data show that HCWs who perceive that they receive more support at work have high CS [32]. Specifically, the bivariate Pearson’s correlations in our study indicated that HCWs’ perceived support at work correlated positively and significantly with their mean perceived CS. These findings imply that access to support may be essential to overcoming the stressful aspects of day-to-day work.

In our study, CS was significantly correlated with age, with people aged 41 years and older having greater CS. Once more, these results align with a recent study’s findings [33]. One explanation that we hypothesize for this result is that the older providers have developed the skills to cope with work demands. Furthermore, independent of clinical experience, maturity and life experience have predicted CS in the literature [32]. However, our research found that years of expertise negatively affected CS: HCWs with 5 or more years of experience had lower CS scores. This is in contrast to the findings of a study which indicated that increased years in the profession were associated with high CS [34]. We hypothesize that our findings concerning the years of expertise could be due to the growing duties and responsibilities that come with more years of experience.

Despite none of the participants having high BO in our study, about half reported an average level of BO, which could be worrisome. These levels are consistent with other studies, which have mainly reported moderate levels of BO [34,35,36]. Interestingly, in our study, gender was not a risk factor for BO, which is inconsistent with research conducted to examine job-related BO among 104 emergency physicians and nurses in Dammam City, SA that reported increased BO in male participants [21]. On the other hand, another study conducted in SA revealed that female psychiatrists and psychiatric trainees scored higher in the BO domain than their male colleagues [22]. These differences in findings could be due to differences in the targeted population (for example, different specialties). Moreover, female psychiatrists have long faced profession-related challenges such as inflexible career structure and being unequally represented compared to their male colleagues [37].

In our results, having children was a protective factor against BO, which is consistent with the results of a study conducted among Israeli burn clinicians that showed that having children decreased the risk of BO and CF, as they may provide emotional support and diversion from work-related stressors [38]. Our study found that physicians were more prone to score higher on the BO domain than other HCWs. Data in the literature are inconsistent regarding the association between BO and the discipline, however. For instance, in a systematic review and meta-analysis exploring BO among different disciplines, no correlation was found between BO and specific disciplines [35]. On the other hand, a study conducted in the US on 764 HCWs found that psychologists and social workers had higher BO than other healthcare providers. As the authors explained, their results could be attributed to psychologists and social workers experiencing more workplace violence [39]. In addition, our figures showed that practitioners with longer working hours (≥61 h/week) had higher BO scores than those with fewer working hours. This finding was consistent with another study conducted in the US targeting nurses working in Texas [34]. We hypothesize that this result could be related to the fact that the more hours that are spent at work, the fewer hours that are available for sleep, leading to sleep deprivation and difficulty achieving a work–life balance. Finally, similarly to previous studies [17,22], our data showed that BO in HCWs correlated negatively with their CS. It might be that increased negative emotions can affect HCWs’ sense of efficacy and thus limit their experience of CS [40].

This research has particular strengths and limitations. Strength-wise, the study addresses a topic that has not been sufficiently studied in SA. Another strength is that the sample was large. A third is that the study was conducted at a national level, including HCWs of all disciplines, including trainees. A fourth strength is that the study used a well-recognized validated scale, namely ProQOL 5.

The study also has certain limitations. First, the study was of a cross-sectional design with a convenience sampling method. Second, as the targeted population included all HCWs, we were limited in obtaining responses from certain groups, such as social workers, as they are primarily Arabic speakers. Future studies with more rigorous research designs and targeting non-English-speaking HCWs (e.g., by using an Arabic questionnaire/scale) are therefore warranted. Lastly, as our study lacks data on the number of hospitals and their respective regions where the participants worked, future studies could provide insights into the representativeness of the sample across different hospitals.

Given the topic’s significance and its associated negative impact that can lead to severe psychological consequences, including anxiety, depressive symptoms, and substance abuse [41], the research team recommends conducting more comprehensive awareness campaigns so that HCWs become more aware of—and informed about—programs and resources that could help them develop better-coping skills and problem-solving techniques. Hence, identifying such resources to whom HCWs can reach out is critical. It is also worth mentioning that self-care is supported as a preventative strategy for work-related stress and BO. Aspects of self-care involving flexibility, physical health, social support, and spiritual practice can help prevent negative consequences and promote well-being [42]. Evidence-based tools are also needed when assessing and treating CF-related symptoms, such as ProQOL 5, Maslach Burnout Inventory and Self-Care Behavior Inventory [41]. Notably, mindfulness, cognitive behavioral therapy, and acceptance and commitment therapy have strong evidence to alleviate such symptoms [41].

## 5. Conclusions

This study aimed to examine the prevalence of CF and its predictive factors among HCWs in SA, which could subsequently aid in promoting a healthier environment for them inside and outside work. The results, including the significant positive correlation between BO and STS and the finding that physicians were more prone to BO than other HCWs, indicate the need for more preventive interventions and awareness campaigns aiming to support HCWs in general, and physicians in particular, to minimize their risk of developing BO and STS and increase their chance of experiencing CS.

## Figures and Tables

**Table 1:** Descriptive analysis of the healthcare workers’ sociodemographic characteristics. *n* = 678.

Sociodemographic	Frequency	Percentage
Sex		
Female	429	63.3
Male	249	36.7
Age		
Younger than 30	324	47.8
30–40 years	259	38.2
41–50 years	67	9.9
Older than 50	28	4.1
Marital status		
Never married	332	49
Ever married	346	51
Having children		
No	404	59.6
Yes	274	40.4
Nationality		
Saudi	505	74.5
Non-Saudi	173	25.5
Residence		
Central region	421	62.1
Western region	55	8.1
Eastern region	132	19.5
Southern region	26	3.8
Northern region	44	6.5
History of psychiatric illness		
No	598	88.2
Yes	80	11.8
History of chronic medical illnesses		
No	541	79.8
Yes	137	20.2
Professional discipline		
Other allied healthcare worker	86	12.7
Nurse	151	22.3
Physician (MD, MBBS)	441	65
Hospital level		
Primary care	155	22.9
Secondary hospital	175	25.8
Tertiary hospital	348	51.3
Service type		
Governmental	616	90.9
Private	62	9.1
Years of clinical experience		
0–2 years	259	38.2
>2–5 years	182	26.8
>5–10 years	90	13.3
More than 10 years	147	21.7
Worked hours per week		
Less than 40 h	154	22.7
41–60 h	420	61.9
61–80 h	73	10.8
More than 80 h	31	4.6
Number of patients treated per week		
Less than 10 patients per week	112	16.5
10–20 patients per week	188	27.7
21–50 patients per week	214	31.6
More than 50 patients per week	164	24.2

**Table 2:** Descriptive analysis of the healthcare workers’ perceptions of general life and work satisfaction and compassion fatigue.

Variable	Mean (SD)	Mean Rank *
General Life and Work Satisfactions		
1. To which degree do you feel supported at your working institution?	2.95 (1.26)	4
2. How do you rate your job satisfaction?	3.16 (1.14)	2
3. How do you rate your personal life satisfaction?	3.18 (1.15)	1
4. How do you rate your financial income satisfaction?	3.00 (1.20)	3
Burnout Indicators		
1. I am happy.	3.54 (0.90)	2
4. I feel connected to others.	3.48 (1.01)	3
8. I am not as productive at work because I am losing sleep over traumatic experiences of a person I help.	2.11 (1.07)	10
10. I feel trapped by my job as a helper.	2.55 (1.19)	9
15. I have beliefs that sustain me.	3.14 (1.18)	6
17. I am the person I always wanted to be.	3.42 (1.04)	4
19. I feel worn out because of my work as a helper.	3.01 (1.10)	8
21. I feel overwhelmed because my case (work) load seems endless.	3.23 (1.06)	5
26. I feel “bogged down” by the system.	3.11 (1.16)	7
29. I am a very caring person.	4.02 (0.93)	1
Compassion Satisfaction Indicators		
3. I get satisfaction from being able to help people.	4.14 (0.93)	1
6. I feel invigorated after working with those I help.	3.30 (1.05)	10
12. I like my work as a helper.	3.78 (0.98)	3
16. I am pleased with how I am able to keep up with [helping] techniques and protocols.	3.51 (0.97)	8
18. My work makes me feel satisfied.	3.47 (1.04)	9
20. I have happy thoughts and feelings about those I help and how I could help them.	3.70 (0.94)	5
22. I believe I can make a difference through my work.	3.67 (0.98)	6
24. I am proud of what I can do to help.	4.00 (0.93)	2
27. I have thoughts that I am a “success” as a helper.	3.55 (0.96)	7
30. I am happy that I chose to do this work.	3.73 (1.06)	4
Secondary Traumatic Stress Indicators		
2. I am preoccupied with more than one person I help.	3.34 (0.96)	1
5. I jump or am startled by unexpected sounds.	2.66 (1.16)	3
7. I find it difficult to separate my personal life from my life as a helper.	2.82 (1.12)	2
9. I think that I might have been affected by the traumatic stress of those I help.	2.34 (1.10)	7
11. Because of my helping, I have felt “on edge” about various things.	2.64 (1.15)	4
13. I feel depressed because of the traumatic experiences of the people I help.	2.36 (1.09)	6
14. I feel as though I am experiencing the trauma of someone I have helped.	2.32 (1.10)	9
23. I avoid certain activities or situations because they remind me of frightening experiences of the people I help.	2.32 (1.14)	8
25. As a result of my helping, I have intrusive, frightening thoughts.	2.28 (1.10)	10
28. I can’t recall important parts of my work with trauma victims.	2.50 (1.00)	5

* Mean rank = the ascending order of the mean score, where greater scores denote greater perception.

**Table 3:** Descriptive analysis of the healthcare workers’ overall perceived compassion fatigue concepts and perceived compassion fatigue indicator levels.

Variable	Mean (SD)	Frequency (%)		
		Low	Average	High
Perceived compassion satisfaction score/level	36.86 (7.22)	19 (2.8)	477 (70.4)	182 (26.8)
Perceived burnout score	23.40 (4.66)	288 (42.5)	390 (57.2)	0
Perceived secondary traumatic stress score	25.57 (6.99)	230 (33.9)	433 (63.9)	15 (2.2)

**Table 4:** Bivariate Pearson’s correlations between the healthcare workers’ perceptions of work related compassion fatigue and other related factors.

Variable	CS	BO	STS	SUP	JS	PLS
Perceived compassion satisfaction (CS) score	1					
Perceived burnout (BO) score	−0.665 **					
Perceived secondary traumatic stress (STS) score	−0.098 *	0.455 **				
Perceived received support at work (SUP) score	0.356 **	−0.418 **	−0.153 **			
Job satisfaction (JS) rating score	0.466 **	−0.445 **	−0.151 **	0.694 **		
Personal life satisfaction (PLS) score	0.403 **	−0.488 **	−0.173 **	0.412 **	0.492 **	
Satisfaction with income rating score	0.268 **	−0.291 **	−0.102 **	0.386 **	0.430 **	0.410 **

** Correlation is significant at the 0.01 level (2-tailed). * Correlation is significant at the 0.05 level (2-tailed).

**Table 5:** Multivariable linear regression analysis of the healthcare workers’ perceived compassion satisfaction score.

Variable	Coefficients Unstandardized Beta	CI for Beta Coefficient 95.0%		*p*-Value
		Lower Bound	Upper Bound	
(Constant)	51.517	48.443	54.592	<0.001
Age ≥ 41 years	1.253	0.540	1.967	0.001
Sex = Male	−0.202	−0.986	0.583	0.614
Marital state = Ever married	−0.661	−1.581	0.258	0.158
Experience years ≥ 5 years	−0.696	−1.172	−0.221	0.004
Perceived support at work score	−0.368	−0.789	0.052	0.086
Perceived job satisfaction level score	1.498	1.022	1.973	<0.001
Perceived burnout score	−1.069	−1.170	−0.967	<0.001
Perceived secondary traumatic stress score	0.254	0.194	0.315	<0.001

Dependent outcome variable= healthcare workers’ perceived compassion satisfaction score. Model overall significance: f(8,669) = 96.75, *p*-value < 0.001. Model R-squared = 0.536, Adj. R-squared = 0.531.

**Table 6:** Multivariable linear regression analysis of the healthcare workers’ perceived work-related burnout score.

Variable	Coefficients Unstandardized Beta	CI for Beta Coefficient 95.0%		*p*-Value
		Lower Bound	Upper Bound	
(Constant)	30.696	28.971	32.420	<0.001
Age group ≥ 41 years	0.305	−0.046	0.657	0.089
Sex = Male	0.170	−0.276	0.616	0.455
Nationality = Saudi	0.425	−0.123	0.973	0.128
Have children = Yes	−0.542	−1.082	−0.002	0.049
Working discipline = Physician	0.350	0.045	0.654	0.024
Region of residence	0.169	−0.003	0.342	0.054
worked weekly hours ≥ 61 h	0.623	0.324	0.923	<0.001
Work department	−0.027	−0.057	0.002	0.072
Prior diagnosis with mental/psychiatric illness = Yes	0.943	0.280	1.606	0.005
Perceived support level at workplace score	−0.367	−0.556	−0.178	<0.001
Perceived secondary traumatic stress score	0.229	0.198	0.260	<0.001
Perceived compassion satisfaction score	−0.343	−0.375	−0.311	<0.001
Perceived personal life satisfaction score	−0.634	−0.845	−0.422	<0.001

Dependent outcome variable = healthcare workers’ perceived work-related burnout score. Model overall significance: f(13,664) = 99.61, *p*-value < 0.001. Model R-squared = 0.661, adj R-squared = 0.654.

**Table 7:** Multivariable linear regression analysis of the healthcare workers’ perceived work related traumatic secondary stress score.

Variable	Unstandardized Beta Coefficients	CI for Beta Coefficient 95.0%		*p*-Value
		Lower Bound	Upper Bound	
(Constant)	−13.485	−19.134	−7.836	<0.001
Age group	−0.136	−0.992	0.720	0.754
Sex = Male	−0.149	−1.076	0.778	0.753
Have children = Yes	0.837	−0.343	2.018	0.164
Residence = Southern provinces	3.460	1.147	5.772	0.003
Residence = Northern provinces	2.393	0.572	4.214	0.010
Working discipline	−0.550	−1.205	0.105	0.100
Weekly work hours	−0.479	−1.132	0.173	0.150
Hospital level	0.441	−0.131	1.013	0.131
Experience years	0.479	−0.102	1.061	0.106
Perceived compassion satisfaction score	0.368	0.285	0.452	<0.001
Perceived burnout score	1.063	0.933	1.192	<0.001

Dependent outcome variable = HCW’s perceived traumatic secondary stress score. Model overall significance: f(11,666) = 27.94, *p*-value < 0.001. Model R-squared = 0.316, adj R-squared = 0.304.

## Data Availability

Data will be made available upon reasonable request directed to the corresponding author.
